# *Monascus* Yellow Pigments Ameliorate Hyperuricemia via Dual Mechanisms: Xanthine Oxidase Inhibition and Uric Acid Transporter Modulation (ABCG2, URAT1, and GLUT9)

**DOI:** 10.3390/foods14162765

**Published:** 2025-08-08

**Authors:** Furong Xue, Renqin Zhu, Jiaxing Li, Zheng Liu, Lidan Niu, Wei Chen, Chengtao Wang, Jie Zheng

**Affiliations:** 1 Key Laboratory of Geriatric Nutrition and Health, Beijing Engineering and Technology Research Center of Food Additives, School of Food and Health, Beijing Technology and Business University, No.33 Fucheng Road, Haidian District, Beijing 100048, China; 2330202165@st.btbu.edu.cn (F.X.); 2330202193@st.btbu.edu.cn (R.Z.); 13253621162@163.com (J.L.); 2330201008@st.btbu.edu.cn (Z.L.); weichen@btbu.edu.cn (W.C.); 2 Key Laboratory of Condiment Supervision Technology for State Market Regulation, School of Chemistry and Chemical Engineering, Chongqing Institute for Food and Drug Control, Chongqing University, No.55 Daxuecheng South Rd, Shapingba, Chongqing 401331, China; niulidan@cqifdc.org.cn

**Keywords:** *Monascus*, xanthine oxidase, organic anion transporters, gastrointestinal microbiome, gut microbiota, oxidative stress

## Abstract

The increasing global prevalence of hyperuricemia (HUA), particularly among younger populations, underscores the urgent need for safe and effective dietary interventions. *Monascus* fungi, long utilized in East Asian food culture, ferment rice to produce red yeast rice (RYR), a functional food rich in monacolin K and Monascus pigments. Among these, *Monascus* yellow pigments (MYPs)—natural azaphilone compounds used as food additives and colorants—have shown antioxidant, anti-inflammatory, and metabolic regulatory activities. However, their potential to alleviate hyperuricemia remains unexplored. This study investigates the urate-lowering and organ-protective effects of MYPs through a combination of in vitro, in vivo, and gut microbiota analyses. MYPs exhibited significant xanthine oxidase (XOD) inhibitory activity, and molecular docking confirmed that monascin (MS) and ankaflavin (AK) competitively bind to the XOD active site. In a murine HUA model, MYPs significantly reduced serum uric acid (SUA) levels without causing hepatic or renal toxicity. Mechanistically, MYPs downregulated renal UA reabsorption transporters (URAT1, GLUT9) and upregulated the excretory transporter ABCG2, enhancing uric acid (UA) excretion. These findings highlight MYPs as promising food-derived bioactives with dual XOD inhibition and uricosuric effects, offering a novel nutraceutical strategy for hyperuricemia prevention and management.

## 1. Introduction

Hyperuricemia, defined as elevated serum uric acid (SUA) concentrations exceeding 420 μmol/L in men and 350 μmol/L in women, is a major metabolic disorder and a key risk factor for gout [1]. In recent decades, it has become the second most prevalent metabolic condition globally after diabetes mellitus, with increasing incidence among younger individuals. In China, the prevalence is estimated at 16.4%, with an upward trend in early-onset cases [2]. In the United States, data from the National Health and Nutrition Examination Survey (NHANES) 2015–2016 reported a prevalence of HUA of 20.2% in men and 20.0% in women [3]. In Korea, NHANES data reported a prevalence of HUA of 11.4% [4]. Beyond gout, hyperuricemia is closely associated with chronic kidney disease [5], cardiovascular disease [6], metabolic syndrome, and increased all-cause mortality [7]—necessitating the development of safe, long-term interventions.

UA is a poorly water-soluble heterocyclic end product of purine metabolism. Unlike most mammals, humans lack the uricase gene, precluding the conversion of UA into the more soluble allantoin; thus, UA must be excreted via the renal and intestinal route [8]. Overproduction of purines or impaired UA excretion results in hyperuricemia [9]. XOD, predominantly expressed in the liver and intestines, catalyzes the oxidation of hypoxanthine to xanthine and, subsequently, xanthine to UA; upregulation of XOD activity, therefore, contributes to UA overproduction [10]. Approximately 70% of UA is eliminated through the kidneys and the remaining 30% via the intestine [11]. Renal handling of UA is mediated by several transporter proteins, URAT1 and GLUT9, located on the apical and basolateral membranes of proximal tubular cells, respectively, which facilitate UA reabsorption [12], whereas the ATP-binding cassette transporter ABCG2 on the basolateral membrane promotes UA secretion [13]. In the gut, UA excretion depends on both microbial catabolism and specific intestinal transporters. Impaired renal function and dysbiosis of the intestinal microbiota diminish UA clearance, culminating in elevated SUA levels [14]. Consequently, XOD and renal UA transporters represent attractive pharmacological targets for anti-hyperuricemic drug development.

Moreover, hyperuricemia promotes tissue injury through oxidative stress and inflammation, partly via activation of the NLRP3 inflammasome [15,16,17,18]. Dysbiosis of the gut microbiota can further exacerbate UA accumulation and systemic inflammation by disrupting intestinal UA catabolism and barrier function [19,20,21]. To rationally explore the interaction between potential inhibitors and these targets, computational approaches, particularly molecular docking, were employed in this study. This in silico analysis serves as a crucial starting point for identifying and prioritizing candidate compounds with high binding affinity and specificity towards XOD or the relevant transporters, thereby guiding subsequent experimental validation and drug development efforts [22].

Current anti-hyperuricemic drugs (e.g., allopurinol, febuxostat, benzbromarone) exhibit limitations such as adverse hepatic or cardiovascular effects, poor patient tolerance, or narrow therapeutic windows [23,24,25,26]. Hence, there is a growing interest in identifying functional food ingredients with multi-target urate-lowering and anti-inflammatory effects [27].

*Monascus* species, particularly *Monascus purpureus*, are edible fungi used in traditional East Asian fermentation. Fermentation of steamed rice with *Monascus* produces RYR, a functional food rich in monacolin K, sterols, and a family of polyketide pigments known as *Monascus* pigments (MPs). MPs are widely applied as food colorants and preservatives in meat and seasoning products [28]. Monascin and ankaflavin, the primary bioactive components of MYPs, are azaphilone derivatives characterized by a fused pyranoquinone bicyclic core and a fatty acid side chain, with documented antioxidant, anti-inflammatory, lipid-lowering, and metabolic regulatory effects [29]. MYPs have also attracted attention in the cosmetics and textile-dyeing industries for their safety and bioactivity [30].

Despite these properties, their potential to modulate uric acid metabolism has not been systematically evaluated. Previous studies have hinted at the serum UA-lowering effects of monascin, but the mechanisms for this remain unclear [31]. Moreover, the impact of MYPs on renal urate transporters, oxidative stress markers, inflammation, and gut microbiota in the context of hyperuricemia has not been addressed.

Therefore, in this study, we comprehensively evaluate the hypouricemic and organ-protective effects of MYPs through xanthine oxidase inhibition, modulation of UA transporters, anti-inflammatory and antioxidant activity, and gut microbiota regulation in a mouse model of hyperuricemia. This work aims to provide a mechanistic basis for the development of MYPs as functional food components or nutraceutical agents for hyperuricemia management.

## 2. Materials and Methods

### 2.1. Material and Reagents

Monascus rice (Batch No. FJ2023-MR0815; production date: August 2024; expiry date: August 2025; stored at 4 °C) was obtained from Gutian Hongqu Company (Gutian Hongqu Company, Gutian, China); XOD, xanthine, and allopurinol were sourced from Shanghai Macklin Biochemical Co., Ltd. (Shanghai, China). Hypoxanthine (HX) and potassium oxonate (PO) (98% purity) were purchased from Beijing Innokeys Technology Co., Ltd. (Beijing, China). Silica gel (100–200 mesh) and paraformaldehyde (CAS No. 30525-89-4) were supplied by Shanghai Aladdin Biochemical Technology Co., Ltd. (Shanghai, China). ELISA kits for mouse IL-6 (FF-ED20188 ), IL-1β (FF-ED20174 ), and TNF-α (FF-ED20852 ) were provided by Fine Biological Technology Co., Ltd. (Nanjing, China). Commercial assay kits for SUA, serum creatinine (CRE), XOD, catalase (CAT), total superoxide dismutase (T-SOD), alanine aminotransferase (ALT), aspartate aminotransferase (AST), and malondialdehyde (MDA) were acquired from Nanjing Jiancheng Bioengineering Institute (Nanjing, China). Kits for blood urea nitrogen (BUN) and glutathione peroxidase (GSH-Px/GPx) were purchased from Beijing Solarbio Science & Technology Co., Ltd. (Beijing, China). Anti-β-actin monoclonal antibody was sourced from Wuhan Boster Biological Technology Co., Ltd. (Wuhan, China), and antibodies against URAT1 (Proteintech, 14937-1-AP), GLUT9 (Abcam, ab223470), ABCG2 (Proteintech, 27286-1-AP), NLRP3 (Proteintech, 68102-1-Ig), caspase-1 (Abcam, ab179515), ASC (Abcam, ab309497), and β-actin ASC (Affinity, Affinity), were obtained from Wuhan Sanying Biotechnology Co., Ltd. (Wuhan, China). All other chemicals were of analytical grade. Microplate reader (BIO-Tek, MQX200); electrophoresis power supply (Liuyi Instrument Plant, Beijing, China, DYY-7C); qPCR system (BIO-RAD, CFX96).

### 2.2. Preparation of Monascus Yellow Pigments

The extraction and separation of *Monascus* yellow pigments were performed using an improved protocol according to Zhou et al. [32]. Briefly, *Monascus* rice powder was extracted with absolute ethanol (1:20 *m*/*v*) under ultrasound-assisted extraction conditions (200 W, 30 min, 50 °C) to obtain the crude pigment extract. This extract was then filtered, concentrated under reduced pressure at 50 °C, and freeze-dried. The resultant concentrate was subjected to silica gel column chromatography (100–200 mesh) for pigment purification. The yellow pigment fractions were eluted using a gradient of n-hexane/ethyl acetate (8:2, *v*/*v*). The eluate was concentrated under vacuum at 50 °C and freeze-dried to obtain the purified MYPs.

### 2.3. In Vitro XOD Inhibition Assay

The XOD inhibitory activity was measured spectrophotometrically using a modified established method [33]. Reaction mixtures comprised phosphate buffer (pH 7.5), xanthine substrate (5 × 10^−5^ mol/L), and MYPs (5, 10, 25, 50, 75, and 100mg/mL) at various concentrations (Table 1). After pre-incubation at 37 °C for 20 min, a pre-warmed XOD solution (3 U/mL) was added. Absorbance at 293 nm was recorded every 1.5 s for 30 s, and the reaction rate (d (A/dt)) was determined from the linear portion of the absorbance–time curve. Inhibition (%) was calculated as

Inhibition (%) = [(1 − A/B) × 100]

where A (Sample group–Blank group) and B (Positive control group–Negative Control) are the reaction rates with and without MYPs, respectively. IC50 values were derived from dose–response curves.

### 2.4. Molecular Docking

To explore the interactions between Ankaflavin or Monascin and XOD, molecular docking was performed using AutoDock4. The crystal structure of XOD (PDB: 3NVW) was retrieved from the RCSB Protein Data Bank. The two-dimensional structures of MS, AK, allopurinol, and Guanine were downloaded from PubChem and converted to three-dimensional conformations using ChemBio3D Ultra, followed by geometry optimization with Avogadro. The XOD receptor was prepared in AutoDock MGLTools1.5.6 by removing water molecules, adding polar hydrogens, and assigning Gasteiger charges. The docking range was set in the active pocket where the original ligand of the target protein was located (X = 37.8, Y = 20.1, Z = 18.3). Docking calculations yielded binding affinities (Vina scores, kcal/mol). Docking poses and intermolecular interactions were visualized in PyMOL and analyzed with Discovery Studio 2025.

### 2.5. Establishment of Hyperuricemia Mouse Model and Treatment

Male Kunming mice (6–8 weeks old) were procured from SPF (Beijing, China) Biotechnology Co., Ltd., and acclimatized for one week under controlled conditions (22 ± 2 °C, 65 ± 5% humidity, 12 h light/dark cycle). Mice were randomly assigned to six groups (*n* = 5): normal control (NC; 0.5% CMC-Na), model control (MC), allopurinol (AP; 20 mg/kg), low-dose MYPs (MYL; 50 mg/kg), medium-dose MYPs (MYM; 100 mg/kg), and high-dose MYPs (MYH; 200 mg/kg).

**Figure 1:**
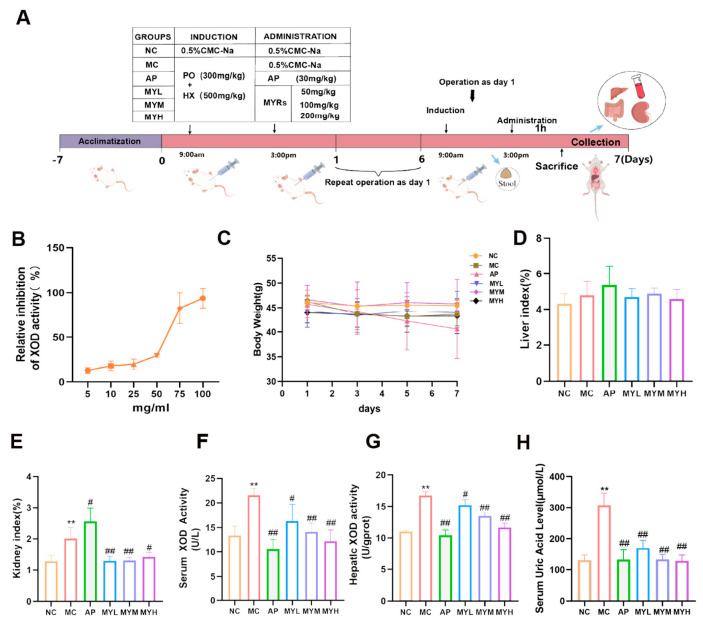
We investigated the systemic effects of *Monascus* yellow pigments (MYPs) on hyperuricemic mice. (NC: normal control; hyperuricemia model; Ap: allopurinol; MYL: 50 mg/kg MYPs; MYM: 100 mg/kg MYPs; MYH: 200 mg/kg MYPs). Animal experimental flowchart (**A**). Effect–concentration curve of MYPs on xanthine oxidase (XOD) activity in vitro (**B**). Body weight changes (**C**). Hepatic and renal functional indices (**D**,**E**). Serum and hepatic XOD activities (**F**,**G**). Serum uric acid (UA) levels (**H**). ** *p* < 0.01 vs. NC group; # *p* < 0.05 vs. HUA group; ## *p* < 0.01 vs. HUA group.

Hyperuricemia was induced using a daily oral gavage of HX (500 mg/kg) and PO (300 mg/kg) for 7 days, while the NC group received 0.5% CMC-Na. Model success was confirmed by measuring SUA levels in randomly selected mice from the NC and MC groups one hour prior to drug administration, as described previously [34]. The treatment groups received MYPs at specified doses, and the NC/MC groups continued with vehicle (Figure 1A). Body weights were recorded on day 0 and every 48 h thereafter. All procedures were approved by the Animal Welfare and Ethics Committee of China Agricultural University (License No.: Aw12402202-5-1; Approval Date: 10 January 2022).

On the final day, fresh feces were collected and stored at −80 °C. One hour after the last dose, blood was drawn from the orbital sinus, allowed to clot (2 h, 25 °C), and centrifuged (1500× *g*, 10 min, 4 °C) to obtain serum. Liver and kidneys were excised, rinsed in cold saline, weighed, and divided; one portion was fixed in 4% paraformaldehyde, one was stored at –80 °C, and one was homogenized (10% *w/v* in 0.9% saline) on ice. Homogenates were centrifuged (12,000× *g*, 15 min, 4 °C), and supernatants were stored at −80 °C.

Organ indices (%) were calculated as

Organ Index (%) = [Organ Weight (g)/Final Body Weight (g)] × 100%

### 2.6. Biochemical Analyses

Serum UA was measured using the phosphotungstic acid reduction method (expressed as μmol/L). XOD activity in serum and liver was assayed by quantifying the hypoxanthine-to-xanthine reaction product at 520 nm using commercial kits (expressed as U/L for serum and U/gprot for liver tissue). Liver function markers (ALT, AST) (expressed as U/gprot) and renal function parameters (BUN, CRE) (expressed as μg/mL for BUN and μmol/L for CRE), along with oxidative stress indicators (CAT, MDA, GSH, SOD) (CAT、MDA、GSH、SODexpressed as U/mL for serum and U/mgprot for renal tissue；MDA expressed as nmol/ml for serum and nmol/mgprot for renal tissue), were assayed according to the manufacturers’ protocols.

### 2.7. Histopathology

Fixed liver and kidney tissues were paraffin-embedded, sectioned (5 μm), dried (65 °C, 6 h), and stained with hematoxylin and eosin (H&E). Images were captured at 200× *g* magnification using a 3DHISTECH Panorama 250 microscope (Debrecen, Hungary).

### 2.8. Cytokine Measurement

Serum levels of IL-1β, IL-6, and TNF-α were quantified using ELISA. Following the manufacturer’s instructions, each sample was assayed in technical triplicate on the same plate. The average optical density values from replicates were used to calculate cytokine concentrations according to the standard curve generated using each assay.

### 2.9. qRT-PCR

Total RNA from kidneys was extracted with the High Purity Total RNA Rapid Extraction Kit (Tiangen Biotech, Beijing, China). cDNA was synthesized using the ReverTra Ace^®^ qPCR RT Master Mix Kit (Tiangen Biotech, Beijing, China). Real-time PCR was performed using the TOYOBO SYBR^®^ Green Realtime PCR Master Mix on a Bio-Rad CFX system. Primer sequences are listed in Table 2. Relative expression was calculated using the 2–∆∆Ct method, using GAPDH as the reference and with triplicate analyses.

### 2.10. Western Blotting

Kidney proteins were extracted and quantified using a BCA assay. Samples (30 μg) were separated on 12% SDS-PAGE, transferred to PVDF membranes, and blocked with 5% skim milk for 1 h. Membranes were incubated overnight at 4 °C, with primary antibodies, washed, and probed with HRP-conjugated secondary antibodies (1:1000 or 1:800) for 1 h. Signals were developed using ECL reagents (BioVision, San Francisco, CA, USA) and imaged on a Bio-Rad Gel Doc 2000 system.

### 2.11. Gut Microbiota Analysis

Fecal DNA was extracted from mice, and then was amplified and sequenced. Operational Taxonomic Units (OTUs) were analyzed using the UPARSE software at 97% similarity. Diversity indices (ACE, Chao, Shannon, and Simpson) were calculated to evaluate species richness, diversity, and coverage within the microbial community. Beta diversity was assessed at the OTU level using principal coordinate analysis (PCoA) and non-metric multidimensional scaling (NMDS). The linear discriminant analysis (LEfSe) method was employed to identify differences in microbial community abundance between groups, with a linear discriminant analysis (LDA) score threshold of 4.0.

### 2.12. Statistical Analysis

All data were analyzed and processed using GraphPad Prism 8.0 software, and all results are expressed as means ± standard deviation (SD). One-way analysis of variance (ANOVA) was applied for the significance of difference calculation of any two groups, and a value of *p* < 0.05 was considered to be a significant difference. Spearman correlation analyses assessed the relationships between microbial taxa and serum TNF-α, IL-1β, and IL-6 levels.

## 3. Results

### 3.1. Effects of MYPs on In Vitro XOD Inhibition and General Parameters in HUA Mice

MYPs exhibited dose-dependent inhibition of XOD in vitro. Plotting inhibition rates against MYP concentrations yielded a regression curve (Figure 1B), from which the IC_50_ was determined to be 61.25 mg/mL via SPSS 27 analysis.

Administration of HX and PO to induce hyperuricemia did not significantly alter body weight trajectories among groups (*p* > 0.05), whereas the AP group exhibited slight weight fluctuation (Figure 1C). Organ indices revealed that both the model and AP groups had significantly elevated kidney coefficients (*p* < 0.01), while liver coefficients remained unchanged (Figure 1D,E). MYPs treatment normalized the kidney index without affecting the liver, indicating renal safety and the contrastive nephrotoxicity of allopurinol. Treatment with allopurinol or MYPs at all doses significantly lowered SUA, with the high-dose MYPs group achieving control-equivalent levels (128.12 ± 19.11 µmol/L). Consistent with UA reductions, hepatic XOD activity was significantly suppressed by all MYP doses (*p* < 0.01) (Figure 1F,G), demonstrating in vivo alignment with in vitro inhibitory effects. MYPs reduce SUA levels and inhibit XOD activity. In vivo hyperuricemic mice displayed a marked rise in SUA (307.43 ± 39.85 µmol/L vs. 129.44 ± 17.35 µmol/L in controls; *p* < 0.01), confirming the model’s validity (Figure 1H).

### 3.2. Molecular Docking Analysis

**Figure 2:**
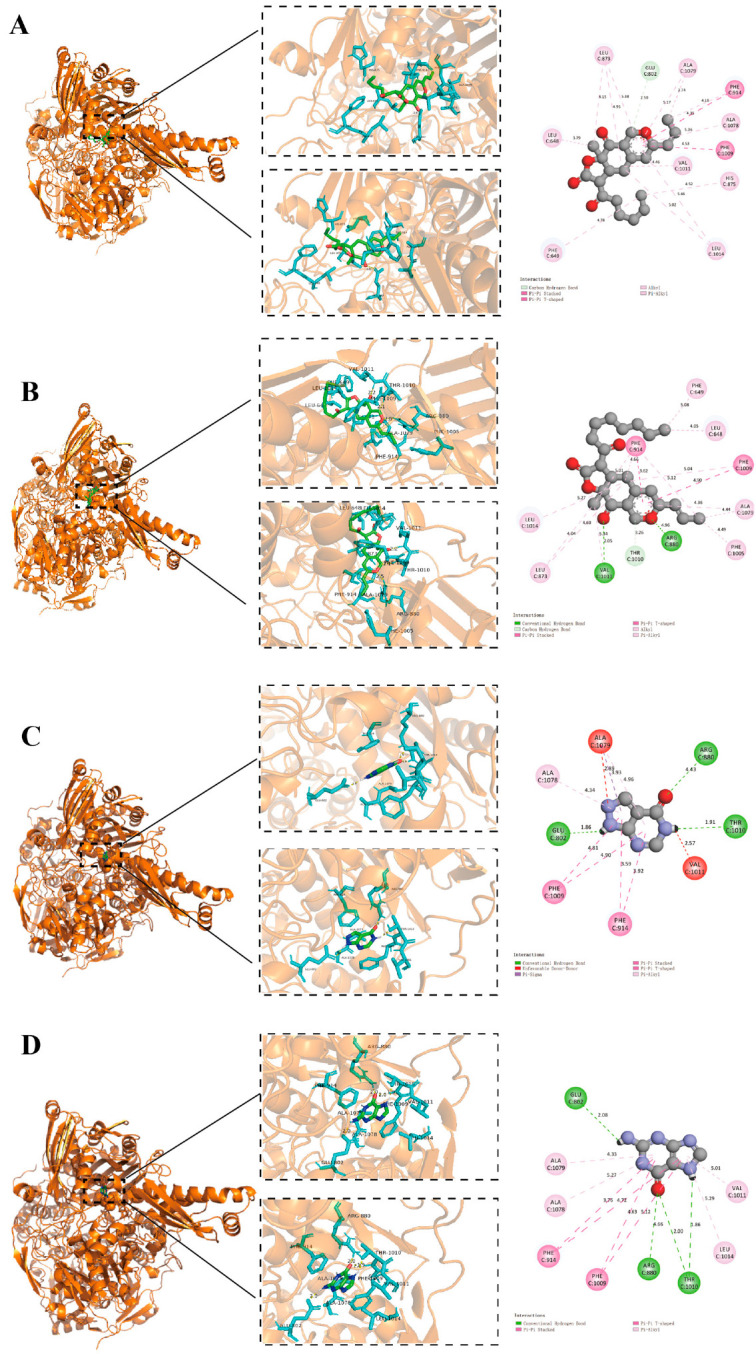
Study on docking modes of MS, AK, AP, and Guanine with Xanthine Oxidase (XOD). (**A**–**D**) Optimal docking conformations of MS, AK, AP, and Guanine (green) within the surface structure of XOD (brown). Three-dimensional and two-dimensional interaction diagrams of each ligand and XOD were generated using AutoDock, with hydrogen bonds highlighted in yellow.

To elucidate the molecular basis of XOD inhibition, AK and MS—the principal MYP components—were docked into the active site of XOD using AutoDock4. Guanine, the native ligand, served as a reference. MS formed hydrogen bonds with GLU-802, achieving a binding energy of −6.2 kcal/mol (Figure 2A), whereas AK interacted with VAL-1011, ARG-880, and THR-1010, yielding −6.63 kcal/mol (Figure 2B). Allopurinol and Guanine shared interactions with THR-1010, ARG-880, and GLU-802, with binding energies of –6.08 kcal/mol and –5.95 kcal/mol, respectively (Figure 2C,D). Additionally, π-π stacking between the aromatic rings of AK/MS and Phe-914/Phe-1009 was observed. These data indicate that AK and MS bind more tightly to XOD than allopurinol, suggesting competitive occupancy of the enzyme’s active pocket and supporting their inhibitory roles.

### 3.3. MYPs Preserve Liver and Renal Function

**Figure 3:**
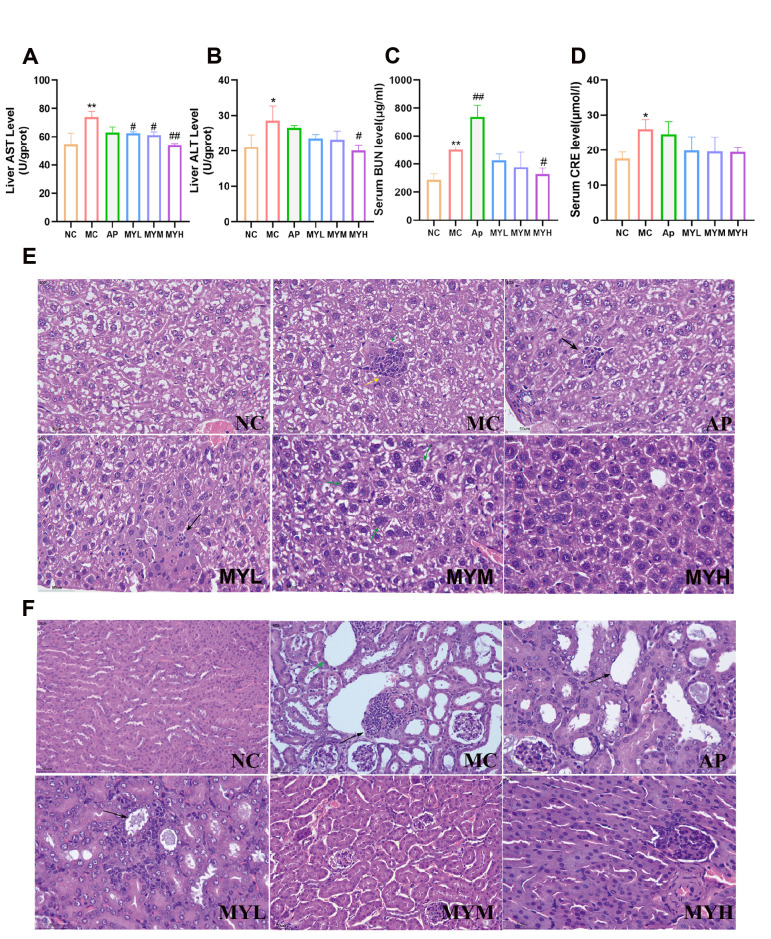
MYPs ameliorated PO- and HX-induced hepatic and renal injury-related biomarker and pathological alterations in HUA mice. (**A**,**B**) Hepatic alanine aminotransferase (ALT) and aspartate aminotransferase (AST) levels. (**C**,**D**)Blood urea nitrogen (BUN) and creatinine (CRE) levels. (**E**,**F**) Representative hematoxylin and eosin (H&E)-stained histological sections of kidney and liver tissues in HUA mice (*n* = 3). In liver sections,green arrows represent scattered binucleate hepatocytes and visible inclusion bodies, black arrows represent hepatocellular necrosis and karyolysis, yellow arrows represent inflammatory cell infiltration; In kidney sections, black arrows represent renal lymphocytic infiltration,green arrows represent renal tubular dilation. Magnification: 200× *g*; * *p* < 0.05 vs. NC group; ** *p* < 0.01 vs. NC group; # *p* < 0.05 vs. HUA group; ## *p* < 0.01 vs. HUA group.

Liver ALT and AST were elevated in the HUA model (*p* < 0.05), but were restored to near-normal values by MYPs, particularly at 200 mg/kg (Figure 3A,B). H&E staining revealed focal granulomas and inflammatory infiltrates in the model and AP groups, whereas MYPs-treated livers maintained normal histology (Figure 3E), affirming hepatic safety.

**Figure 4:**
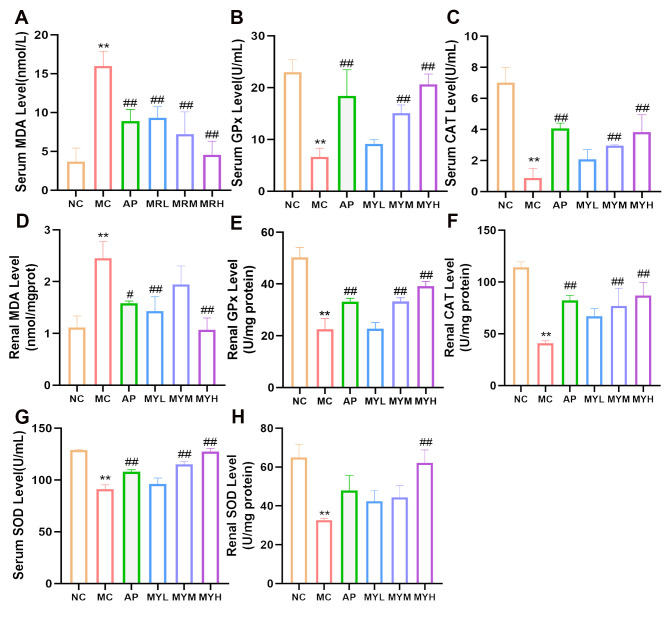
MYPs modulated serum and renal oxidative stress biomarkers in mice. (**A**) Serum malondialdehyde (MDA) levels. (**B**) Serum glutathione peroxidase (GPx) levels. (**C**) Serum catalase (CAT) levels. (**D**) Renal MDA levels. (**E**) Renal GPx levels. (**F**) Renal CAT levels. (**G**) Serum superoxide dismutase (SOD) levels. (**H**) Renal SOD levels. ** *p* < 0.01 vs. NC group; # *p* < 0.05 vs. HUA group; ## *p* < 0.01 vs. HUA group.

MYPs alleviate hyperuricemia-induced significant increases in serum BUN and CRE (*p* < 0.05); Figure 3c–d). in serum BUN and CRE (*p* < 0.05; Figure 3C,D). High-dose MYPs reduced serum BUN significantly (*p* < 0.05) and tended to lower serum CRE, while allopurinol exacerbated BUN levels (*p* < 0.01). The observed exacerbation of BUN levels by allopurinol may be related to its inhibition of xanthine oxidase, potentially leading to the accumulation of poorly soluble xanthine/hypoxanthine precursors, which could contribute to renal tubular injury [35]. Histopathology showed tubular dilation and inflammatory infiltrates in the model and AP groups; MYPs dose-dependently ameliorated these lesions (Figure 4C–H), underscoring superior renal safety.

### 3.4. MYPs Alleviate Oxidative Stress in Hyperuricemic Mice

Oxidative stress contributes to the pathogenesis of HUA through ROS accumulation, which damages cellular components and activates the NLRP3 inflammasome. We quantified antioxidant enzyme activities (SOD, CAT, GPx) and the lipid peroxidation marker MDA in serum and renal tissues (Figure 4). PO- and HX-induced HUA significantly decreased SOD, CAT, and GPx activities while elevating MDA levels (*p* < 0.05 vs. NC). Treatment with allopurinol and medium-/high-dose MYPs (MYM, MYH) restored these enzyme activities and reduced MDA in both compartments (*p* < 0.05 vs. MC). Low-dose MYPs (MYL) significantly reduced MDA (*p* < 0.05), but did not significantly affect SOD, CAT, or GPx. Notably, MYH more effectively recovered SOD activity and decreased MDA than AP (*p* < 0.05), demonstrating the superior attenuation of oxidative stress.

### 3.5. MYPs Restore Renal Uric Acid Transporter Expression

**Figure 5:**
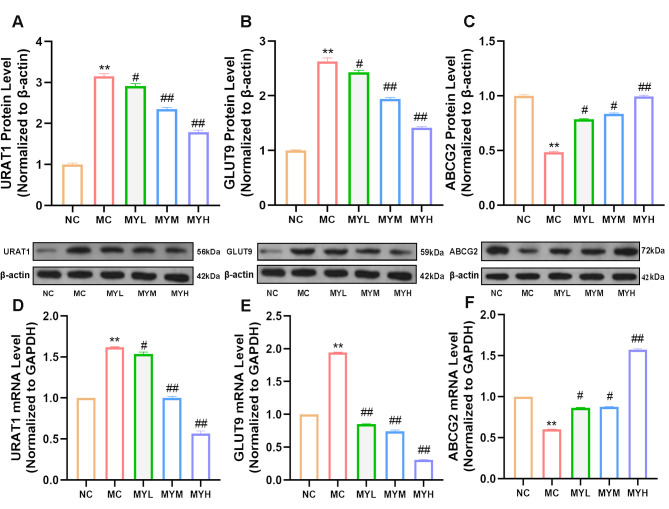
MYPs regulated renal urate transporter protein and mRNA expression in hyperuricemic mice. (**A**–**C**) Western blot analysis showing relative protein expression levels of URAT1, GLUT9, and ABCG2 in kidney tissues across groups. (**D**–**F**) qPCR results showing relative mRNA expression levels of URAT1, GLUT9, and ABCG2 in kidney tissues. ** *p* < 0.01 vs. NC group; # *p* < 0.05 vs. HUA group; ## *p* < 0.01 vs. HUA group.

Renal UA excretion depends on the coordinated activity of reabsorption transporters URAT1 and GLUT9 and the secretion transporter ABCG2 [36]. Western blotting revealed that HUA upregulated URAT1 and GLUT9 protein expression and downregulated ABCG2 (*p* < 0.01 vs. NC; Figure 5A). MYP treatment dose-dependently reversed these alterations, suppressing URAT1/GLUT9 and enhancing ABCG2 levels (*p* < 0.01 vs. MC). qRT-PCR confirmed these protein-level changes at the transcript level, with MYPs normalizing URAT1, GLUT9, and ABCG2 mRNA expression (*p* < 0.01 vs. MC; Figure 5B), indicating promoted UA clearance.

### 3.6. MYPs Attenuate Hyperuricemia-Induced Inflammation

**Figure 6:**
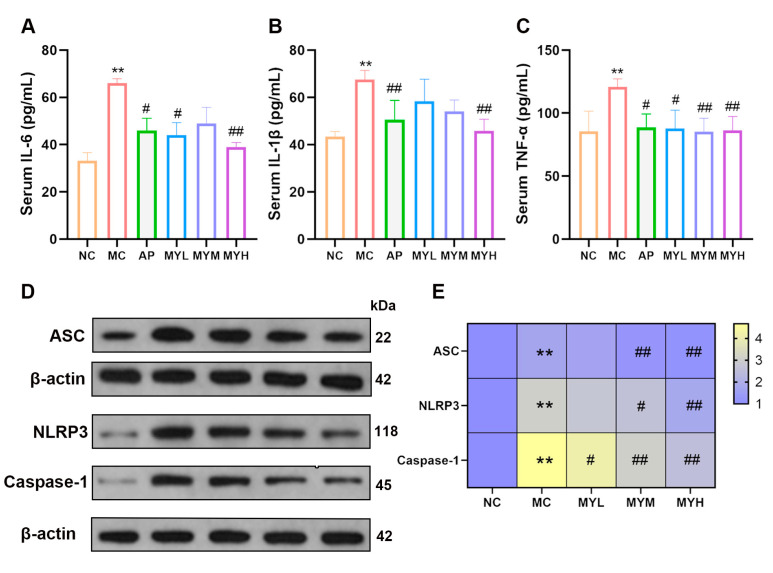
MYPs suppressed inflammatory cytokines and NLRP3-mediated inflammasome signaling. (**A**–**C**) ELISA quantification of serum inflammatory cytokine levels: interleukin-6 (IL-6), interleukin-1β (IL-1β), and tumor necrosis factor-α (TNF-α). (**D**,**E**) Western blot (WB) analysis of NLRP3, apoptosis-associated speck-like protein containing a CARD (ASC), and caspase-1 protein expression in renal tissues. ** *p* < 0.01 vs. NC group; # *p* < 0.05 vs. HUA group; ## *p* < 0.01 vs. HUA group.

Hyperuricemia triggers systemic inflammation through ROS-mediated NF-κB activation and peroxidase inhibition, elevating TNF-α, IL-6, and IL-1β [37]. ELISA assays showed that PO/HX increased serum IL-6, TNF-α, and IL-1β (*p* < 0.05 vs. NC; Figure 6A–C). Both AP and high-dose MYPs (MYH) significantly reduced these cytokines (*p* < 0.05 vs. MC), with MYH exhibiting the most pronounced anti-inflammatory effect.

MYPs suppress NLRP3 inflammasome activation. The NLRP3 inflammasome, comprising NLRP3, ASC, and caspase-1, underlies IL-1β maturation in HUA [16]. Renal expression of NLRP3, ASC, and caspase-1 proteins was elevated in HUA mice (*p* < 0.05 vs. NC; Figure 6D–E). MYPs reduced these proteins in a dose-dependent manner, with MYH achieving reductions of 33.1% (ASC), 26.4% (NLRP3), and 45.8% (caspase-1) (*p* < 0.01 vs. MC), suggesting the direct inhibition of inflammasome activation.

### 3.7. MYPs Modulate Gut Microbiota in Hyperuricemic Mice

**Figure 7:**
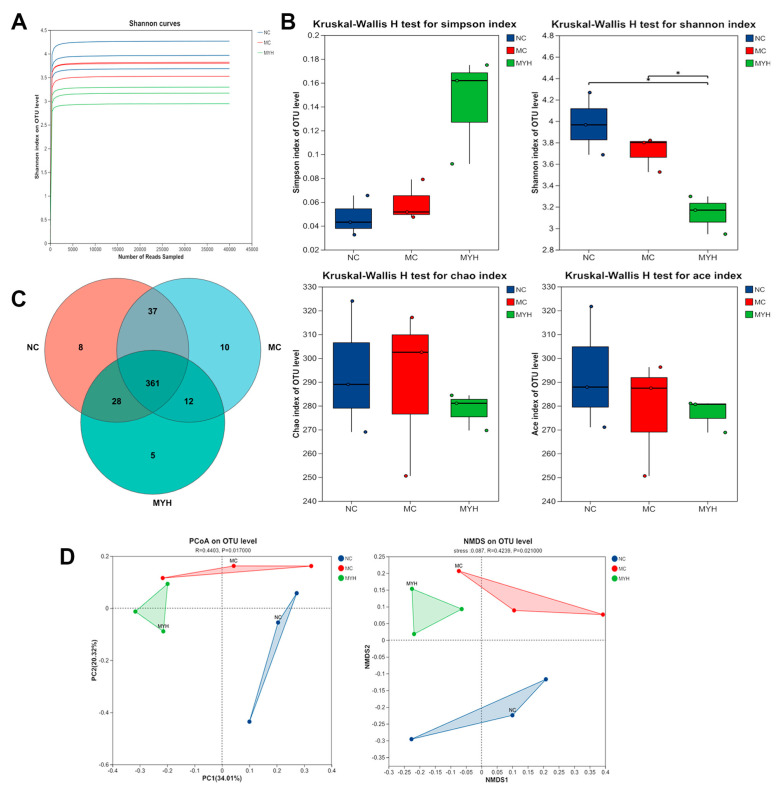
Effects of MYPs on gut microbiota composition and function. (**A**) Rarefaction curves of operational taxonomic units (OTUs) clustered at 97% sequence similarity across groups. (**B**) Alpha diversity indices: Ace, Chao, Shannon, and Simpson. (**C**) Venn diagram of OTU distribution. (**D**) Beta diversity analysis using principal coordinate analysis (PCoA) and non-metric multidimensional scaling (NMDS). * *p* < 0.05. .

Gut microbiota dysbiosis contributes to HUA via impaired barrier function and systemic inflammation [19,38]. 16S rRNA sequencing of fecal samples from NC, MC, and MYH groups showed plateaued rarefaction curves (Figure 7A) and OTU counts of 434, 420, and 406, respectively (Figure 7C). Alpha diversity (Chao, ACE, Shannon, Simpson) indicated reduced richness and diversity in MC mice, partially restored by MYH (*p* < 0.05 for Shannon index; Figure 7B). PCoA and NMDS demonstrated distinct community structures among groups (Figure 7D).

**Figure 8:**
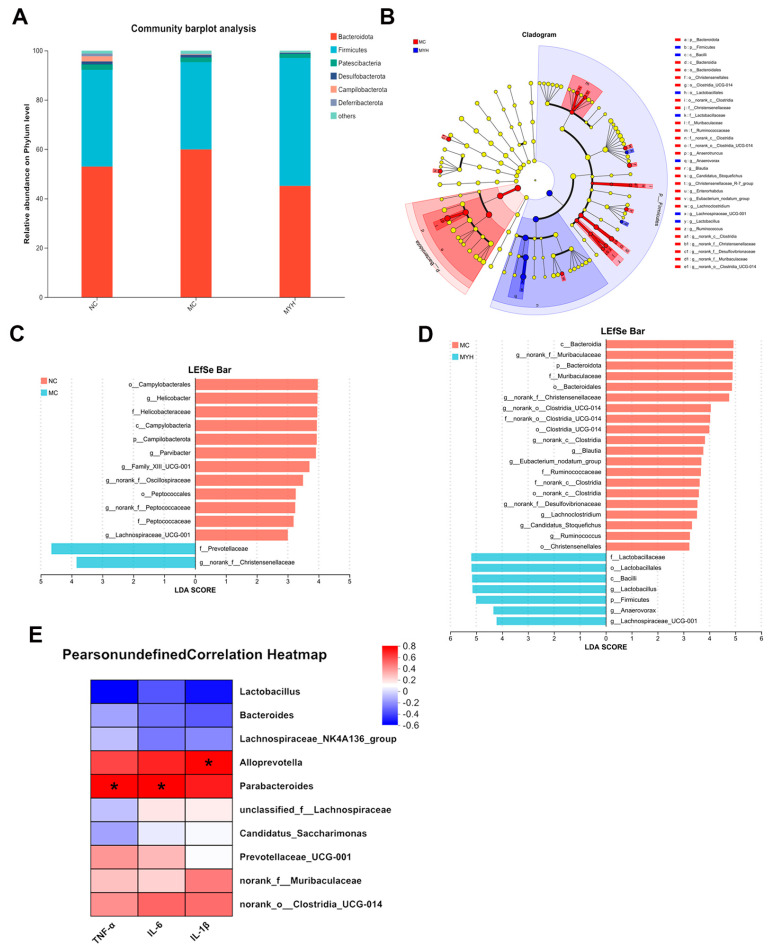
Effects of MYPs on gut microbiota composition and function . (**A**) Relative abundance of bacterial phyla in gut microbiota. (**B**) Phylogenetic cladograms of microbial communities. (**C**–**E**) Linear discriminant analysis effect size (LEfSe) comparison of cecal bacteria between (**C**) NC and HUA groups and (**D**) HUA and MYH (200 mg/kg MYPs) groups. (**E**) Spearman correlation analysis between gut microbiota composition and serum inflammatory cytokines (IL-1β, TNF-α, IL-6). * *p* < 0.05 vs. NC group.

At the phylum level, HUA increased the Firmicutes/Bacteroidota ratio (Figure 8A). LEfSe analysis (LDA > 4) identified pro-inflammatory taxa enrichment (*Christensenellaceae*, *Lachnoclostridium*, *p_Firmicutes*) in MC mice and anti-inflammatory taxa (*Campylobacterales*, *Helicobacter*) in NC mice (Figure 8B–D). MYH reversed these shifts, decreasing *Christensenellaceae* and *Firmicutes* while enriching *Bacteroidota*, *Lactobacillaceae*, and *Lactobacillus*, which are associated with SCFA production and anti-inflammatory activity. MYH also reduced *Muribaculaceae*, *Ruminococcus*, and *Anaerovorax*, suggesting the restoration of the UA metabolism. Spearman correlation analysis linked *Alloprevotella* and *Parabacteroides* positively with TNF-α, IL-6, and IL-1β, while *Lactobacillus* correlated negatively (*p* < 0.05; Figure 8E). These microbiota alterations coincide with reduced cytokine levels, indicating that MYPs ameliorate HUA by reshaping the gut microbiota and disrupting the inflammation axis.

## 4. Discussion

The global burden of HUA is increasing at an alarming rate, and is now considered a major public health issue due to its association with a spectrum of metabolic and inflammatory disorders, including gout, hypertension, chronic kidney disease (CKD), and cardiovascular diseases [39]. Notably, the prevalence of HUA is no longer confined to middle-aged or elderly populations—it is emerging as a concern among younger individuals, particularly adolescents. Recent epidemiological data show a 23% increase in the incidence of HUA among Chinese adolescents, reflecting shifting dietary habits, increased consumption of purine-rich foods (animal offal, sea fish, beer, high-sugar foods, etc.), sedentary lifestyles, and genetic susceptibility [40,41]. Despite its growing prevalence, therapeutic options for managing HUA remain limited. Clinically approved urate-lowering drugs, such as xanthine oxidase (XO) inhibitors (e.g., allopurinol, febuxostat) and uricosurics (e.g., benzbromarone, probenecid), are associated with adverse effects, including hypersensitivity reactions, hepatotoxicity, and nephrotoxicity, necessitating the development of safer, more effective alternatives [42].

Given that the safety of natural substances is crucial for health, an increasing number of studies are now exploring the development of natural substances targeting HUA. Natural substances, such as green tea polyphenols [43], fucoidan [15], and water-soluble lemon extracts [44], reduce uric acid levels by inhibiting uric acid synthesis, promoting its excretion, and protecting renal function.

This study presents a comprehensive investigation of MYPs as a potential multi-targeted natural therapeutic against HUA. Derived from *Monascus* spp., MYPs are a group of polyketide compounds traditionally consumed in Asian fermented foods such as red yeast rice. Among these, MS and AK have gained attention for their antioxidant, anti-inflammatory, and hypolipidemic properties [28]. However, their potential anti-hyperuricemic activities have not been systematically evaluated until now. Our findings highlight the multifaceted effects of MYPs on UA metabolism, oxidative stress, inflammatory signaling, renal urate transport, and gut microbiota composition, positioning them as promising nutraceutical candidates with both preventive and therapeutic utility in HUA.

XOD catalyzes the oxidation of hypoxanthine to xanthine and subsequently to UA, playing a central role in purine catabolism and UA biosynthesis. XOD inhibitors such as allopurinol have long been first-line therapies for HUA and gout [45]; however, their use is sometimes limited by toxicity or hypersensitivity. Our in vitro data reveal that MYPs exert a significant inhibitory effect on XOD activity, with an IC_50_ of 61.25 mg/mL.

Molecular docking simulations provided mechanistic insights into this inhibition. Both MS and AK demonstrated favorable binding affinities to the XOD active site, interacting with key amino acid residues involved in substrate stabilization and electron transfer. These findings suggest a competitive inhibition mode, whereby MS and AK hinder uric acid production by occupying the catalytic pocket of the enzyme. These interactions mirror those of known XO inhibitors, confirming the potential of these natural pigments to serve as leads for further drug development or functional food formulations aimed at reducing UA biosynthesis. MYPs are composed of a structurally diverse group of azaphilone derivatives, of which MS and AK are the most well characterized. However, the presence of other minor constituents in the pigment matrix may contribute to the observed pharmacological effects, either individually or synergistically. The present study utilizes MS and AK as representative compounds for molecular docking simulations, providing mechanistic insights into their interaction with xanthine oxidase. Nevertheless, we acknowledge that this does not fully capture the complexity of the extract. Future work incorporating chromatographic profiling (e.g., HPLC, LC-MS), chemical fingerprinting, and activity-guided fractionation will be critical to elucidate the specific roles and interactions of individual components within the MYP matrix.

To validate the therapeutic potential of MYPs, we established a hyperuricemic mouse model by administering a combination of PO—a uricase inhibitor—and HX—a purine precursor that boosts UA synthesis. This dual approach induces rapid and consistent elevation of serum UA levels, mimicking the pathophysiological conditions of human HUA more accurately than single-agent models [46]. MYPs administered orally at 200 mg/kg for 7 days significantly lowered serum UA concentrations from 307.43 ± 39.85 µmol/L in model animals to 128.12 ± 19.11 µmol/L—comparable to the effects of allopurinol (131.83 ± 32.33 µmol/L).

Importantly, liver and kidney function markers, including ALT, AST, CRE, and BUN, remained within physiological ranges following MYPs treatment. This suggests that MYPs exert XO-inhibitory and urate-lowering effects without the hepatorenal toxicity commonly associated with conventional pharmacological agents. Such safety is critical for the long-term management of chronic metabolic conditions like HUA. Approximately 60–70% of daily uric acid excretion occurs via the kidneys. Renal handling of UA involves a complex interplay of reabsorptive and secretory transporters. Among them, URAT1 (SLC22A12) and GLUT9 (SLC2A9) facilitate UA reabsorption in the renal proximal tubules, while ABCG2, an efflux transporter, promotes UA excretion into the renal tubules and intestines [47].

Our data show that MYPs administration significantly downregulated URAT1 and GLUT9 protein expression levels, reducing reabsorption of UA, while upregulating ABCG2, enhancing its renal and extrarenal excretion. This dual modulation of urate transport resembles the mechanism of action of uricosuric agents like benzbromarone, but without the associated hepatotoxicity. Furthermore, this action addresses the pathogenic root in approximately 60% of HUA patients, who exhibit impaired UA excretion rather than overproduction [36,48]. These findings underscore MYPs’ potential as a uricosuric agent in addition to its XO-inhibitory effect, supporting a dual-action therapeutic profile.

Hyperuricemia contributes to systemic oxidative stress and chronic low-grade inflammation, which in turn exacerbate endothelial dysfunction, nephropathy, and cardiovascular injury [49]. Excessive UA acts as a pro-oxidant by activating NADPH oxidase and mitochondrial ROS generation pathways, leading to the depletion of endogenous antioxidant defenses [50]. MYPs treatment restored the activities of key antioxidant enzymes—SOD, CAT, and GPx—in serum and kidney tissues. Concurrently, MYPs significantly lowered MDA levels, a marker of lipid peroxidation, indicating the mitigation of oxidative stress. These results are consistent with previous studies demonstrating the antioxidative properties of MS and AK, which are known to activate Nrf2-mediated antioxidant pathways [51].

**Figure 9:**
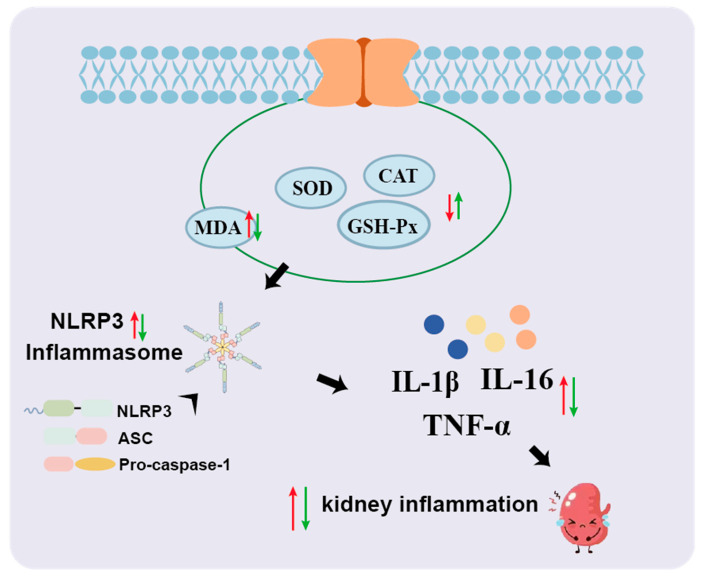
MYPs alleviate hyperuricemia and tissue damage by inhibiting inflammatory cytokines and NLRP3 signaling. Red arrows denote acute pathological alterations induced by HUA in murine models, while green arrows indicate the ameliorative trends of these inflammatory markers following MYPs intervention.

Inflammation is another hallmark of HUA-induced tissue damage. Elevated serum levels of TNF-α, IL-6, and IL-1β are commonly observed in HUA and gout patients, reflecting inflammasome activation and immune dysregulation [52]. Our study shows that MYPs significantly reduced the expression of these cytokines, suggesting an anti-inflammatory role. Moreover, MYPs treatment inhibited the NLRP3 inflammasome pathway, as evidenced by decreased expression of NLRP3, ASC, and caspase-1 in kidney tissues. This pathway is critically involved in UA-induced sterile inflammation and the development of gouty arthritis. By suppressing NLRP3 activation, MYPs may mitigate UA-driven organ injury and inflammatory signaling, adding to their protective profile (Figure 9).

Emerging research has revealed a bidirectional relationship between gut microbiota and uric acid metabolism. Intestinal flora not only contribute to purine degradation and uric acid excretion, but also modulate host immune responses and metabolic homeostasis [53]. Dysbiosis—a disruption in microbial composition—is commonly observed in HUA patients and is associated with systemic inflammation and metabolic dysfunction [54]. 16S rRNA gene sequencing in our study revealed that MYPs intervention led to a notable reshaping of the gut microbiota, reducing the relative abundance of *Bacteroidota*, a phylum enriched in HUA-associated dysbiosis, while enhancing Firmicutes, which are often depleted in metabolic disorders.

LEfSe analysis further identified a significant enrichment of *Lactobacillusspecies*—well-known probiotics capable of producing short-chain fatty acids (SCFAs)—inhibiting XOD, and maintaining gut barrier integrity [55]. Although the present study did not directly quantify SCFAs, the observed enrichment of *Lactobacilluscorrelates* with established reports of its metabolic functions [56]. Prior studies have demonstrated that Lactobacillus-dominant microbiota profiles, such as those induced here by MYPs, are associated with elevated fecal SCFA levels (particularly acetate and butyrate) and reduced systemic inflammation [57]. Additionally, MYPs suppressed the abundance of *Christensenellaceae.* The inverse correlation between *Lactobacillusenrichment* and inflammatory markers (e.g., TNF-α, IL-1β) suggests potential mediation by microbial metabolites like SCFAs [28], further supporting the hypothesis that microbiota remodeling contributes to the anti-inflammatory and hypouricemic effects of MYPs. Future studies integrating metabolomics (e.g., quantification of fecal SCFAs, xanthine derivatives) are warranted to directly validate these functional links [58].

While our study provides compelling evidence for the therapeutic potential of MYPs against hyperuricemia, several limitations warrant further exploration. First, the exact contributions of MS and AK to the observed effects remain to be fully elucidated. Although docking and preliminary in vitro data suggest their pivotal roles, isolated compound testing in vivo and in clinical trials is necessary to confirm their individual efficacy and safety. Second, this study was conducted in a mouse model, and translational relevance to human physiology requires validation through well-designed clinical studies. Dose optimization, pharmacokinetics, and long-term safety evaluations are essential for future nutraceutical development. Additionally, the complex composition of MYPs may present challenges around standardization and regulatory approval, necessitating detailed profiling and quality-control protocols. Lastly, our findings open the door to exploring combination therapies, where MYPs may serve as adjuncts to conventional urate-lowering agents, potentially allowing for dose reduction and side-effect mitigation.

## 5. Conclusions

In conclusion, this study elucidates the urate-lowering mechanisms of *Monascus* yellow pigments, involving xanthine oxidase inhibition, renal urate transporter modulation, oxidative stress reduction, inflammasome suppression, and gut microbiota remodeling. Their dual XOD inhibition and uricosuric action, coupled with their established food safety profile, suggest MYPs’ potential role in hyperuricemia management.

However, the preclinical nature and focus on mechanistic exploration represent key limitations. Future research should prioritize confirming their efficacy and safety in human clinical trials and focus on optimizing bioactive components to enhance therapeutic translatability.

## Figures and Tables

**Table 1:** Reaction system for XOD inhibition assay.

Reagent (mL)	GROUPS			
	Sample	Blank	Positive Control	Negative Control
PBS	1.6	1.95	1.75	2.1
MYPs	0.15	0.15	0	0
Xanthin	1.4	1.4	1.4	1.4
XOD	0.35	0	0.35	0

* Volumes (in mL) of each reagent added to the reaction mixtures for different experimental groups. PBS: Phosphate-Buffered Saline; MYPs: *Monascus* Yellow Pigments; XOD: Xanthine Oxidase. Sample: Contains test compound (MYPs) and enzyme (XOD). Blank: Contains test compound (MYPs), but lacks enzymes (XOD); used to correct for background absorbance of the sample. Negative Control: Lacks both test compound (MYPs) and enzymes (XOD); represents baseline reaction.

**Table 2:** Primer sequences for RT-qPCR.

Gene	Forward Primer (5′–3′)	Reverse Primer (5′–3′)
GAPDH	GGCAAGTTCAACGGCACAG	CGCCAGTAGACTCCACGACAT
URAT1	CTCCATGCTGTGCTGGTTTG	CACAATCCCGATGAGTGCCT
GLUT9	CGGCTCTTCTAACCGTCACA	ACGGAAACATGGGCTTTCTGA
ABCG2	CCATCCAACAGGCCTAGAATCA	TCCTAGGAAGGCCGTTGTTG
		

* Forward and reverse primer sequences (from 5′ to 3′) for target genes and the housekeeping gene glyceraldehyde-3-phosphate dehydrogenase (‘GAPDH’). Gene abbreviations: URAT1 (Urate Transporter 1), GLUT9 (Glucose Transporter 9), ABCG2 (ATP-Binding Cassette Subfamily G Member 2).

## Data Availability

The original contributions presented in this study are included in the article. Further inquiries can be directed to the corresponding authors.

## Abbreviations

The following abbreviations are used in this manuscript:

ABCG2	ATP-Binding Cassette Subfamily G Member 2
AK	Ankaflavin
ALT	Alanine Aminotransferase
AP	Allopurinol
ASC	Apoptosis-Associated Speck-Like Protein Containing a CARD
AST	Aspartate Aminotransferase
BUN	Blood Urea Nitrogen
Caspase-1	Cysteine Aspartate Protease 1
CAT	Catalase
CKD	Chronic Kidney Disease
CMC-Na	Carboxymethyl Cellulose Sodium
CRE	Creatinine
ELISA	Enzyme-Linked Immunosorbent Assay
GAPDH	Glyceraldehyde-3-Phosphate Dehydrogenase
GLUT9	Glucose Transporter 9
GPx/GSH-Px	Glutathione Peroxidase
H&E	Hematoxylin and Eosin
HX	Hypoxanthine
IL-1β	Interleukin-1 Beta
IL-6	Interleukin-6
LPS	Lipopolysaccharide
MC	Model Control
MDA	Malondialdehyde
MOPs	*Monascus* Orange Pigments
MPs	*Monascus* Pigments
mRNA	Messenger Ribonucleic Acid
MYPs	*Monascus* Yellow Pigments
MYH	High-dose *Monascus* Yellow Pigments
MYM	Medium-dose *Monascus* Yellow Pigments
MYL	Low-dose *Monascus* Yellow Pigments
MRPs	*Monascus* Red Pigments
MS	*Monascin*
NC	Normal Control
NF-κB	Nuclear Factor Kappa-Light-Chain-Enhancer of Activated B Cells
NLRP3	NOD-, LRR- and Pyrin Domain-Containing Protein 3
PBS	Phosphate-Buffered Saline
PO	Potassium Oxonate
qRT-PCR	Quantitative Real-Time Polymerase Chain Reaction
SOD	Superoxide Dismutase
SUA	Serum Uric Acid
TNF-α	Tumor Necrosis Factor Alpha
UA	Uric Acid
URAT1	Urate Transporter 1
WB	Western Blot
XOD	Xanthine Oxidase
